# Administration of Phosphonate Inhibitors of Dehydrogenases of 2-Oxoglutarate and 2-Oxoadipate to Rats Elicits Target-Specific Metabolic and Physiological Responses

**DOI:** 10.3389/fchem.2022.892284

**Published:** 2022-06-20

**Authors:** Victoria I. Bunik, Artem V. Artiukhov, Alexey V. Kazantsev, Vasily A. Aleshin, Alexandra I. Boyko, Alexander L. Ksenofontov, Nikolay V. Lukashev, Anastasia V. Graf

**Affiliations:** ^1^ Department of Biokinetics, A. N. Belozersky Institute of Physicochemical Biology, Lomonosov Moscow State University, Moscow, Russia; ^2^ Department of Biochemistry, Sechenov University, Moscow, Russia; ^3^ Faculty of Bioengineering and Bioinformatics, Lomonosov Moscow State University, Moscow, Russia; ^4^ Faculty of Chemistry, Lomonosov Moscow State University, Moscow, Russia; ^5^ Faculty of Biology, Lomonosov Moscow State University, Moscow, Russia

**Keywords:** 2-oxoglutarate dehydrogenase, 2-oxoadipate dehydrogenase, DHTKD1, phosphonate analog of 2-oxo acid, regulation of brain metabolism

## Abstract

*In vitro* and in cell cultures, succinyl phosphonate (SP) and adipoyl phosphonate (AP) selectively target dehydrogenases of 2-oxoglutarate (OGDH, encoded by *OGDH/OGDHL*) and 2-oxoadipate (OADH, encoded by *DHTKD1*), respectively. To assess the selectivity in animals, the effects of SP, AP, and their membrane-penetrating triethyl esters (TESP and TEAP) on the rat brain metabolism and animal physiology are compared. Opposite effects of the OGDH and OADH inhibitors on activities of OGDH, malate dehydrogenase, glutamine synthetase, and levels of glutamate, lysine, citrulline, and carnosine are shown to result in distinct physiological responses. ECG is changed by AP/TEAP, whereas anxiety is increased by SP/TESP. The potential role of the ester moiety in the uncharged precursors of the 2-oxo acid dehydrogenase inhibitors is estimated. TMAP is shown to be less efficient than TEAP, in agreement with lower lipophilicity of TMAP *vs*. TEAP. Non-monotonous metabolic and physiological impacts of increasing OADH inhibition are revealed. Compared to the non-treated animals, strong inhibition of OADH decreases levels of tryptophan and beta-aminoisobutyrate and activities of malate dehydrogenase and pyruvate dehydrogenase, increasing the R–R interval of ECG. Thus, both metabolic and physiological actions of the OADH-directed inhibitors AP/TEAP are different from those of the OGDH-directed inhibitors SP/TESP, with the ethyl ester being more efficient than methyl ester.

## Introduction

The *DHTKD1*-encoded 2-oxoadipate dehydrogenase (OADH) is a member of the family of the thiamine-diphosphate-dependent dehydrogenases of 2-oxo acids, whose well-known representative is 2-oxoglutarate dehydrogenase, encoded by *OGDH* and *OGDHL* genes. Both OADH and OGDH function within their multienzyme complexes (OADHC and OGDHC, correspondingly). Apart from OADH or OGDH, the complexes include the two other common components, the *DLST*-encoded dihydrolipoamide succinyl transferase and *DLD*-encoded dihydrolipoyl dehydrogenase, with multiple copies of all the enzymatic components self-assembled into a complex (Bunik, 2017). Although both OADH and OGDH catalyze oxidative decarboxylation of 2-oxoadipate, the function of OADH is not redundant. Hereditary dysfunctions of OADH cause disease states, such as impaired insulin sensitivity (Xu et al., 2018; Xu et al., 2019) or Charcot–Marie-Tooth disease (Xu et al., 2012; Xu et al., 2018; Luan et al., 2020; Castro-Coyotl et al., 2021; Yalcintepe et al., 2021). Large-scale genome association studies reveal that common variants of the human *DHTKD1* gene may cause type 2 diabetes and cardiovascular problems (Wu et al., 2014; Plubell et al., 2018; Timmons et al., 2018; Wang et al., 2021). These findings highlight physiological importance of the differences in the 2-oxoadipate saturation of the OADH and OGDH isoenzymes, characterized in the native enzyme complexes enriched from mammalian tissues (Artiukhov et al., 2021) and in the complexes reconstituted from their components expressed in *E.coli* (Nemeria et al., 2017; Nemeria et al., 2018).

**FIGURE 1:**
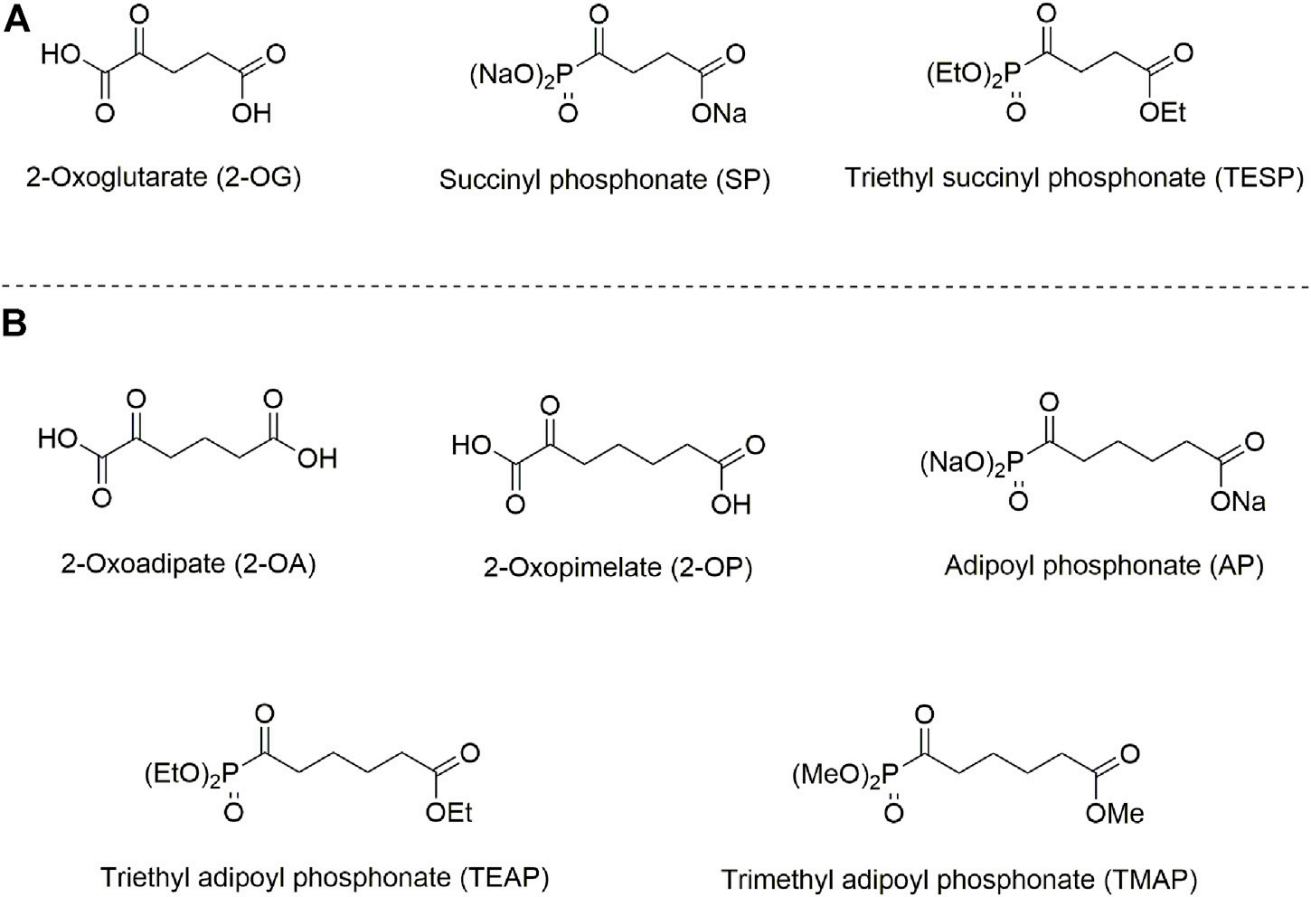
Structures of 2-oxo acids, their phosphonate analogs, and esters, used in this study. **(A)** 2-Oxoglutarate and its phosphonate analog succinyl phosphonate (SP) and triethyl succinyl phosphonate (TESP). **(B)** 2-Oxoadipate and its homolog 2-oxopimelate and the phosphonate analog of 2-oxopimelate adipoyl phosphonate (AP), triethyl adipoyl phosphonate (TEAP), and trimethyl adipoyl phosphonate (TMAP).

The isoenzyme-directed inhibitors may help in a better understanding of specific physiological impacts of the isoenzymes and molecular mechanisms of the isoenzyme-dependent pathologies. In its turn, this understanding is needed to successfully fight the diseases. However, selective pharmacological inhibition of structurally similar isoenzymes *in vivo* is a challenge. Even if inhibitors prefer specific isoenzymes *in vitro,* the drug metabolism, intracellular distribution, and different levels of the isoenzyme expression, etc., may perturb the selectivity *in vivo*. In our recent work, specific action of homologous phosphonate analogs of 2-oxoglutarate and 2-oxoadipate on OADH and OGDH has been characterized in kinetic experiments employing isolated complexes of the isoenzymes (Artiukhov et al., 2021) and in cultured cells (Artiukhov et al., 2020). Using available structural data (Bezerra et al., 2020; Leandro et al., 2020), the enzyme determinants of this specific action are revealed (Artiukhov et al., 2021). Due to spatial constraints in the isoenzyme active sites, the phosphonate analog of 2-oxopimelic acid (adipoyl phosphonate, AP), which is one methylene group longer than the OADH substrate 2-oxoadipate, may still be accommodated by OADH but does not fit into the active site of OGDH. Thus, the phosphonate analogs of the OGDH substrate 2-oxoglutarate and the OADH substrate homolog 2-oxopimelate, shown in Figure 1, are tested in the current work as the isoenzyme-specific inhibitors *in vivo*. The goal of this work was to characterize selectivity of the OGDH- and OADH-directed phosphonates and relative efficiency of their esters upon administration of the inhibitors to animals.

## Materials and Methods

### Reagents

**SCHEME 1:**
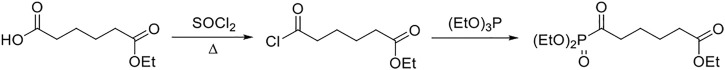
Synthesis of triethyl ester of adipoyl phosphonate (TEAP) from monoethyl adipate using thionyl chloride (SOCl_2_) and triethyl phosphite (EtO)_3_P.

Syntheses of triethyl ester of SP (TESP) and trimethyl ester of AP (TMAP) were performed as described previously (Bunik et al., 2005; Artiukhov et al., 2020): TESP was synthesized from triethyl phosphite and ethyl succinyl chloride; TMAP was synthesized from trimethyl phosphite and methyl adipoyl chloride. Trisodium salts of SP and AP were obtained from TESP and TMAP, respectively, via alkaline hydrolysis (Bunik et al., 2005; Artiukhov et al., 2020). Triethyl ester of AP (TEAP) was synthesized from monoethyl adipate according to Scheme 1. A mixture of adipic acid monoethyl ester (17.3 mmol, 3.01 g) and thionyl chloride (21.6 mmol, 2.57 g, 1.6 ml) was refluxed for 2 h. An excess of SOCl_2_ was removed at reduced pressure. Ethyl adipoyl chloride was thus obtained and used without further purification. Triethyl phosphite (1.05 equiv., 18.2 mmol, 3.02 g, 3.1 ml) was added to ethyl adipoyl chloride at 0°C. The resulting mixture was stirred at ambient temperature for 18 h. The product (ethyl 6-(diethoxyphosphoryl)-6-oxohexanoate, TEAP) was isolated by vacuum distillation as a colorless oil. Yield: 3.65 g (71.7%), b.p. 145–148°C/0.3 mm. NMR ^1^H (CDCl_3_), δ, ppm: 4.14 (m, 4H, (CH_3_CH_2_O)_2_P(O)), 4.04 (q, *J* 7.2 Hz, 2H, COOCH_2_CH_3_), 2.78 (t, *J* 6.6 Hz, 2H, CH_2_C(O)P(O)), 2.23 (m, 2H, CH_2_COOEt), 1.57 (m, 4H, CH_2_CH_2_CH_2_CH_2_), 1.29 (t, *J* 7.1 Hz, 6H, (CH_3_CH_2_O)_2_P(O)), and 1.17 (t, *J* 7.2 Hz, 3H, COOCH_2_CH_3_). NMR ^13^C (CDCl_3_), δ, ppm: 210.6 (d, *J* 167.3 Hz, C(O)P(O)), 173.0 (COOEt), 63.6 (d, *J* 7.0 Hz, (CH_3_ CH_2_O)_2_P(O)), 60.2 (COOCH_2_CH_3_), 42.7 (d, *J* 54.2 Hz, CH_2_C(O)P(O)), 33.8 (CH_2_COOEt), 24.0 (CH_2_ CH_2_ CH_2_CH_2_), 16.2 (d, *J* 5.7 Hz, (CH_3_CH_2_O)_2_P(O)), and 14.1 (COOCH_2_ CH_3_). NMR ^31^P (CDCl_3_), δ, ppm: 2.8. The NMR spectra are given in Supplementary Figure S1. IR spectrum (cm^−1^) (Supplementary Figure S2) was registered on a Thermo Scientific Nicolet iS5 FT-IR spectrometer using iD3- attenuated total reflectance (ATR) accessory. IR bands in the range of 2,365–2,340 cm^−1^ belong to atmospheric CO_2_. IR (neat, cm^−1^) *ν* 1734 (C=O), 1,696 (C=O), and 1,256 (P=O). Anal. Calcd. for C_12_H_23_O_6_P: C, 48.98; H, 7.88; P, 10.53; found: C, 48.87; H, 7.75; P, 10.42.

NAD^+^ was obtained from Gerbu (Heidelberg, Germany), and oxidized glutathione—from Calbiochem (La Jolla, United States). All other reagents were of the highest purity available and obtained from Sigma-Aldrich (St. Louis, United States).

### Animal Experiments

Animals were adapted to housing facilities for at least 1 week prior to the beginning of the experiment, at which time they were approximately 8 weeks old. Rats were maintained on a 12-h/12-h light/dark cycle (lights on at 9:00 a.m. and off at 9:00 p.m.), group-housed (typically four to six in each cage, according to (Leary et al., 2020)), and always given free access to water and rodent pellet food (laboratorkorm.ru). All animal procedures described were approved by the Bioethics Committee of Lomonosov Moscow State University (protocol number 69-o from 09.06.2016) and were in accordance with the Guide for the Care and Use of Laboratory Animals published by the European Union Directives 86/609/EEC and 2010/63/EU.

SP, TESP, AP, TEAP, and TMAP were dissolved in saline to 0.2 M or 1M concentration and were administered at 0.02 mmol/kg (all phosphonates) or 0.1 mmol/kg dosage (TMAP only), respectively. The administration was performed intranasally to pass the blood–brain barrier (Graff and Pollack, 2004; Badhan et al., 2014; Djupesland et al., 2014). Physiological solution (0.9% NaCl) was administered to the control animal group. The rats were subjected to physiological tests and sacrificed by decapitation as described before (Aleshin et al., 2020; Aleshin et al., 2021), 24 h after the substance administration. Immediately after the decapitation, the animal brain was excised and the brain cortex was separated on ice and frozen in liquid nitrogen within 90 s after decapitation. The tissue samples were stored at −70°C until the biochemical assays.

### Physiological Tests

The physiological monitoring was performed 24 h after the administration of phosphonates or physiological solution. The “open field” test (Sestakova et al., 2013; Díaz-Morán et al., 2014) was performed in an arena of 97 cm diameter (“OpenScience,” Moscow, Russia). The test was used to quantify animal behavior as described before (Aleshin et al., 2021; Graf et al., 2022). Anxiety level was characterized, based on the duration and number of grooming acts, duration of freezing, and number of defecation acts. Exploratory activity was assessed by the number of rearing acts. Locomotor activity was estimated by the number of line crossings.

ECG was recorded for 3 min using non-invasive electrodes as previously described (Aleshin et al., 2021). Balance of the heart autonomous regulation was assessed by the following parameters of ECG: an average R–R-interval (R–R, ms), standard deviation of R–R (SD, ms), a range of R–R values, i.e., a difference between the maximal and minimal values (dX, ms), root mean square of successive differences in R–R (RMSSD, ms), and stress index (SI, arbitrary units).

### Biochemical Assays

Quantification of low-molecular weight metabolites in the methanol/acetic acid extracts of rat cerebral cortex was performed as described previously (Artiukhov et al., 2022). The extracts were prepared according to Ksenofontov et al. (2017): the brain tissue (0.5 g) was homogenized in 4 ml ice-cold methanol, with the resulting homogenate diluted with 0.2% acetic acid at a 1:1.5 ratio. After shaking and deproteinization by centrifugation, the supernatants were stored at −70°C until analysis. To characterize the amino acid profiles, 50 µl sample aliquots were loaded on the 2622SC-PF sulfopolystyrene cation-exchange column (Hitachi Ltd., P/N 855-4,507, 4.6 mm × 60 mm) and eluted by a series of lithium acetate buffers with post-column derivatization performed using the Ninhydrine Coloring Solution Kit (Wako Pure Chemical Industries, Osaka, Japan), as described in Ksenofontov et al. (2017). The derivatized products were detected spectrophotometrically at 570 and 440 nm. The chromatograms were processed using MultiChrom for Windows software (Ampersand Ltd., Moscow, Russia). Levels of NAD^+^ and oxidized glutathione were measured with previously described fluorometric assays (Artiukhov et al., 2022) in a microplate format using a CLARIOstar Plus microplate reader (BMG Labtech, Ortenberg, Germany).

Enzyme activities were measured spectrophotometrically in cerebral cortex homogenates as described in previous studies (Araujo et al., 2013; Tsepkova et al., 2017; Artiukhov et al., 2022). The brain tissue (0.5 g) was homogenized in 1.25 ml of 50 mM MOPS, pH = 7.0, containing 2.7 mM EDTA, 20% glycerol, and protease inhibitors (0.2 mM AEBSF, 0.16 µM aprotinin, 3.33 µM bestatin, 3 µM E-64, 2 µM leupeptin, and 1.4 µM pepstatin A) using ULTRA-TURRAX^®^ T-10 basic disperser (IKA, Staufen, Germany). The homogenates were sonicated in the Bioruptor^®^ (Diagenode, Liege, Belgium) and mixed with 40 mM Tris–HCl, pH = 7.4, containing 600 mM NaCl, 4 mM EDTA, 1% sodium deoxycholate, and 4% NP-40, at 3:1 ratio at least for 30 min before the enzyme activity assays. The activities of glutamate dehydrogenase, malate dehydrogenase, NADP^+^-dependent malic enzyme, OGDHC, and extramitochondrial OADHC were detected using absorbance of NAD(P)H at 340 nm (Tsepkova et al., 2017). PDHC activity was detected by absorbance of the iodonitrotetrazolium-formazan product at 500 nm (Artiukhov et al., 2022). Glutamine synthetase activity was assayed by absorbance of the γ-glutamilhydoxamate-Fe^3+^ complex at 540 nm after 15 min of the start of the reaction (Levintow, 1954).

### Statistics and Data Analysis

Data are presented as mean ± standard error of mean (SEM) for each experimental group. Heatmaps were prepared in RStudio (RStudio, PBC) with a *pheatmap* package. Clustering of experimental groups and parameters was performed using “Euclidean” as the distance method and “ward.D2” as the agglomeration method. Experimental groups were compared using one-way ANOVA with Tukey’s *post hoc* test, employed in GraphPad Prism 9.0 (GraphPad Software Inc., La Jolla, United States). The differences with *p* ≤ 0.05 were considered significant. Outliers were identified according to iterative Grubb’s test and excluded from further statistical analysis. The number of animals in experimental groups (*n*), indicated in figures, includes outliers shown as hollow points in the graphs.

## Results

### Specific Biochemical and Physiological Effects of SP/TESP and AP/TEAP

**FIGURE 2:**
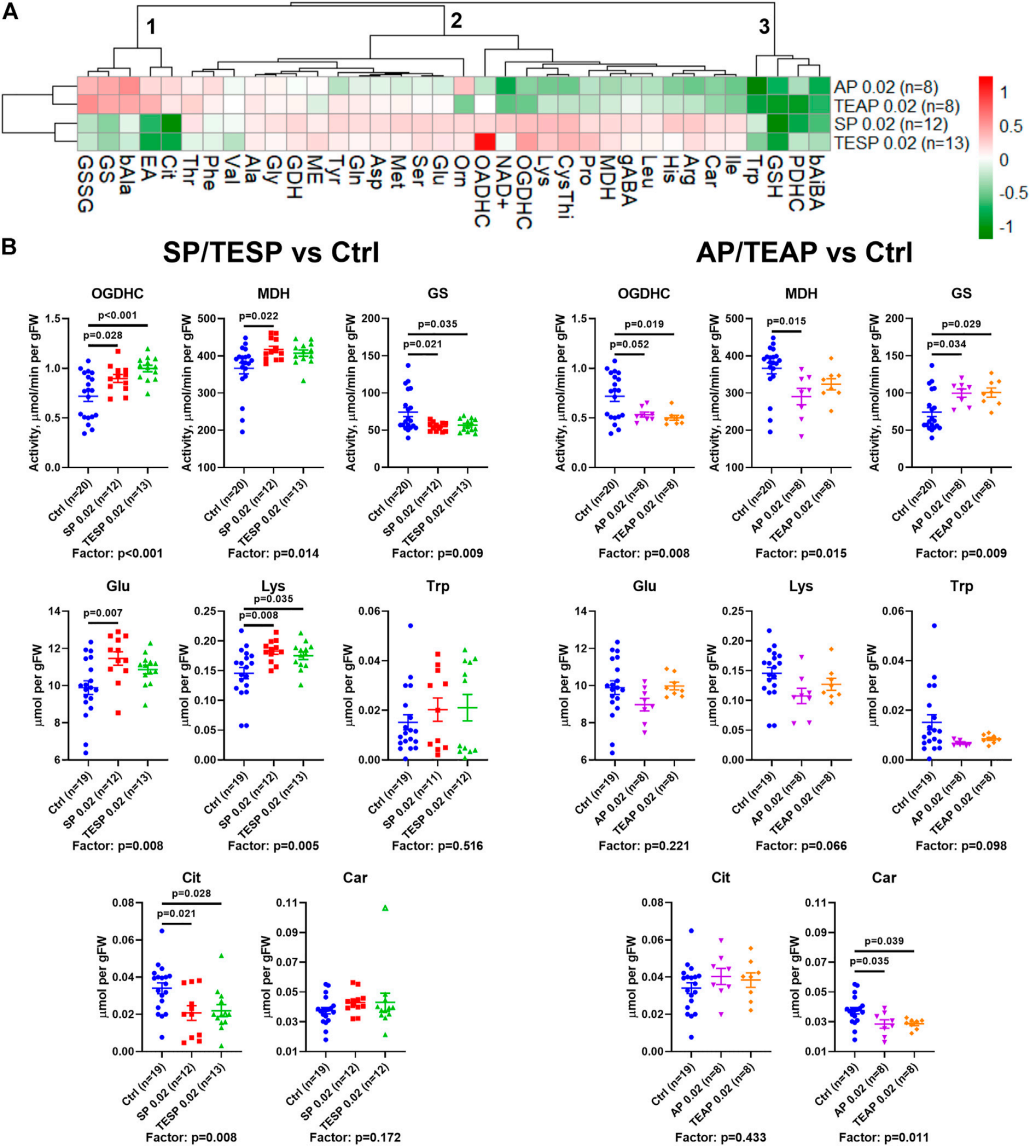
Specific effects of succinyl phosphonate (SP), adipoyl phosphonate (AP), and their triethyl esters (TESP and TEAP, respectively) on biochemical indicators of the OGDH and OADH inhibition in the rat cerebral cortex. All the inhibitors are administered at 0.02 mmol per kg. **(A)** Heatmap of the treatment-induced changes in the levels of metabolites and enzyme activities, presented as log2 of the fold-change from their average levels in the non-treated animals. **(B)** Changes in the absolute values of selected indicators of the OGDH and OADH inhibition after administration of SP/TESP or AP/TEAP, compared to the control values. Significant (*p* ≤ 0.05) differences between the experimental groups, estimated by ANOVA with Tukey’s post hoc test, are indicated as exact *p*-values on the graphs. ANOVA significances of the treatment factor are shown below the graphs. The hollow symbol corresponds to an outlier excluded according to the iterative Grubb’s test. The number of animals in experimental groups (*n*) is indicated including outliers. Proteinogenic amino acids are abbreviated according to the standard three-letter code. Other abbreviations are given in the Glossary section.

Metabolic changes in 36 biochemical parameters of the rat brain, induced by specific inhibitors of OGDHC (SP/TESP) or OADHC (AP/TEAP), are presented as a heatmap shown in Figure 2A. The automatic sorting procedure arranges these metabolic changes into clusters, based on the level of coupling and/or similarity between the different changes. The clusters at the left of Figure 2A reveal strong differences in the metabolic effects of the OGDHC or OADHC inhibitors, along with highly similar actions of the inhibitors directed to one target, i.e., SP and TESP or AP and TEAP. This implies that the two separate clusters of strongly different metabolic changes describe the action of AP/TEAP (upper cluster at the left, Figure 2A) or SP/TESP (lower cluster at the left, Figure 2A). Thus, the clusters of metabolic changes induced by the inhibitors reveal their specific action *in vivo*.

The clusters are shown at the top of Figure 2A to sort out the observed metabolic changes into the three major clusters. Cluster 1 includes the parameters increased by AP/TEAP and decreased by SP/TESP. These changes are inherent in the glutamine synthetase activity and redox-related metabolites, such as disulfide of glutathione, citrulline (a surrogate marker of NO^•^ production), and β-alanine (a precursor of the antioxidant carnosine). Cluster 2 comprises a subcluster with stronger changes, including the activities of OGDHC, malate dehydrogenase, and extramitochondrial OADHC. In this subcluster, metabolic parameters change opposite to those in cluster 1. Finally, cluster three includes four metabolic parameters undergoing decreases in response to both the OGDH and OADH inhibitors. This cluster comprises the levels of tryptophan, glutathione, β-aminoisobutyrate, and the activity of PDHC (Figure 2A). Thus, analysis of the inhibitor-induced metabolic changes reveals that distinct responses of the brain metabolism to SP/TESP and AP/TEAP are associated with the activities of OGDHC, malate dehydrogenase, OADHC, and glutamine synthetase, whereas the commonalities of the metabolic action of SP/TESP and AP/TEAP may be mediated by PDHC.

Statistical analysis of the inhibitor-specific changes in selected indicators from different clusters is presented in Figure 2B. Activities of OGDHC and malate dehydrogenase, essential for the tricarboxylic (TCA) cycle flux, undergo concerted significant changes, increasing in response to SP/TESP and decreasing in response to AP/TEAP. In contrast, the glutamine synthetase activity is significantly decreased by SP/TESP and increased by AP/TEAP. The levels of the amino acids synthesized from 2-oxoglutarate, such as glutamate and lysine, increase upon the OGDHC inhibition but do not significantly change upon the OADHC inhibition. Finally, the redox-state-related indicators citrulline and carnosine manifest specific actions of the OGDH- and OADH-directed inhibitors. Levels of either citrulline or carnosine are significantly decreased by SP/TESP or AP/TEAP, correspondingly.

### SP/TESP Affects Anxiety While AP/TEAP Affects ECG

**FIGURE 3:**
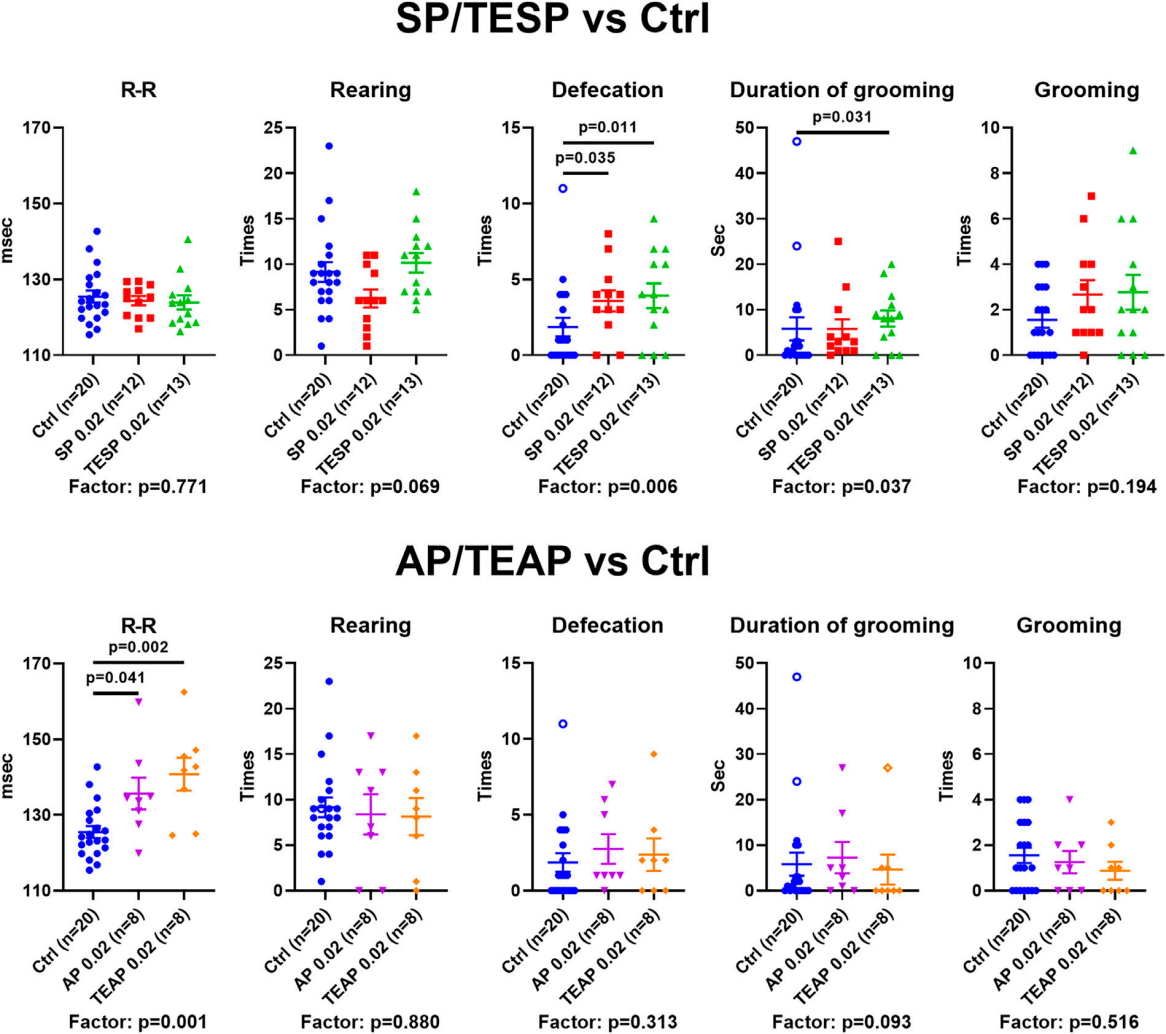
Specific physiological effects of administration of succinyl phosphonate (SP), adipoyl phosphonate (AP), and their triethyl esters (TESP and TEAP) to animals. All inhibitors are administered at 0.02 mmol/kg. R–R interval of ECG, number of rearing acts, number of defecation acts, duration of grooming, and number of grooming acts are shown in the control (Ctrl) animals and those treated with SP/TESP (upper part) or AP/TEAP (lower part). Significant (*p* ≤ 0.05) differences between the experimental groups, estimated by ANOVA with Tukey’s *post hoc* test, are indicated on the graphs. The ANOVA *p*-values for the treatment factor significance are shown below the graphs. The hollow points correspond to outliers excluded from statistical analysis according to the iterative Grubb’s test. The number of animals in experimental groups (*n*) is indicated including outliers.

Specific changes in the brain cortex metabolism, induced by SP/TESP or AP/TEAP (Figure 2), are expected to elicit specific physiological responses. Indeed, different reactivities of selected physiological indicators to administration of the same dose (0.02 mmol/kg) of the inhibitors of OGDH or OADH are shown in Figure 3. Administration of SP or TESP does not affect the R–R interval of ECG but increases anxiety, obvious from the increased number of defecations and duration of grooming (Figure 3, upper part). Administration of AP/TEAP has different effects: the average R–R interval is increased, while the number of defecations and duration of grooming is not affected (Figure 3, lower part). Thus, specific physiological responses to the inhibitors of OGDH or OADH are observed (Figure 3), in good accordance with the inhibitor-specific changes in the brain cortex metabolism (Figure 2).

### Comparison of the Action of the Triethyl and Trimethyl Esters of AP on the Brain Metabolism

According to the previous *in vitro* and cellular studies, the *in vivo* inhibition by the fully esterified phosphonates, which is not observed *in vitro*, is attributed to intracellular hydrolysis by esterases (Bunik et al., 2005), which are abundant in the brain (Mangas et al., 2016; Theilmann et al., 2021). Therefore, the *in vivo* inhibition of OADH by administration of AP esters (Figure 1) is supposed to be due to the characterized inhibitory action of the charged phosphonate AP (Artiukhov et al., 2020; Artiukhov et al., 2021), that is, the inhibitory species released inside cells by esterases. Similar to TESP (Bunik et al., 2005; Zundorf et al., 2009), the fully esterified AP is thus a prodrug. However, the nature of the ester moiety may affect the prodrug delivery and/or formation/action of its partial esters. In fact, in case of partial esters of SP, the phosphonate methyl and ethyl esters are also inhibiting, though to a lower extent than SP itself (Bunik et al., 1992; Biryukov et al., 1996; Bunik et al., 2005). It is therefore interesting to compare *in vivo* action of the AP esters with different ester residues, namely the ethylated TEAP and methylated TMAP (Figure 1).

**FIGURE 4:**
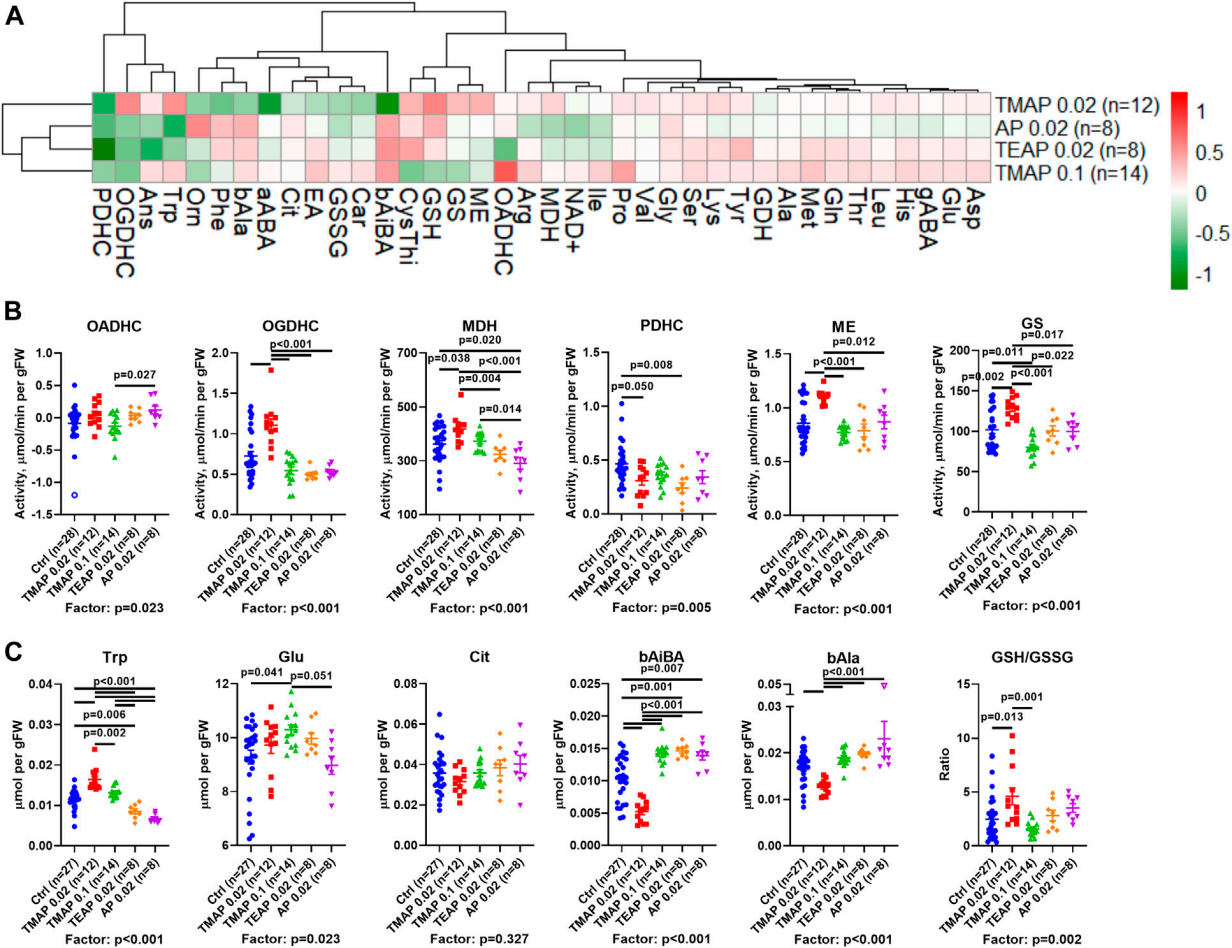
Effects of adipoyl phosphonate (AP) and its trimethyl (TMAP) and triethyl (TEAP) esters on biochemical indicators in the rat cerebral cortex. **(A)** Heatmap of the inhibitor-induced changes in the levels of metabolites and enzymatic activities, presented as log2 of the fold-change in the treated animals, compared to the non-treated ones. **(B)** Statistical analysis of the group differences in the activities of selected enzymes. **(C)** Statistical analysis of the group differences in the levels of metabolites. Significant (*p* ≤ 0.05) differences between the experimental groups, estimated by ANOVA with Tukey’s *post hoc* test, are indicated on the graphs. ANOVA *p*-values for the treatment factor significance are shown below the graphs. Hollow points show outliers according to the iterative Grubb’s test, excluded from statistical analysis. The indicated number of animals in experimental groups (*n*) includes outliers. Proteinogenic amino acids are abbreviated according to the standard three-letter code. Other abbreviations used are given in the Abbreviations section.

Clustering of the metabolic changes induced by the employed treatments (Figure 4A, clusters at the left) combines the effects of the high dose (0.1 mmol/kg) of TMAP with those of the low doses (0.02 mmol/kg) of AP and TEAP. In contrast, the effects of the low TMAP dose (0.02 mmol/kg) form a different subcluster, as some of these effects oppose or are stronger than those of the other treatments (Figure 4). This implies that the absence of an increase or even a reversal of metabolic effects at increasing doses of TMAP (e.g., α-aminobutyric acid (aABA) or β-aminoisobutyric acid (bAiBA) in Figure 4A) is similar to non-monotonous action of increasing doses of TESP (Artiukhov et al., 2022).

A comparison of lipophilicity coefficients (log P, Table 1) indicates that the lipophilicity of TEAP is four-fold higher than that of TMAP, while lipophilicities of TMAP and TESP are similar. Based on these estimations, at a fixed dose (0.02 mmol per kg) of all the compounds, intracellular concentrations of TMAP and TESP should be similar and lower than the intracellular concentration of more lipophilic TEAP. On the other hand, similar action of TMAP and TEAP at a five-fold difference in their dose (Figure 4) roughly corresponds to a four-fold difference in the lipophilicity of these esters (Table 1).

**TABLE 1:** Lipophilic indexes (log P) for the 2-oxo phosphonates and their esters used in this work. The structures are presented in Figure 1. Values of log P are calculated using ChemBioDraw Ultra v. 14.0 (PerkinElmer, Whatham, United States).

Compound name	log P-Value
SP	−0.54
TESP	0.58
AP	0.25
TMAP	0.34
TEAP	1.37

Based on the lipophilicity and dosage, the order of the employed treatments in Figures 4B,C corresponds to increasing intracellular concentrations of the esterified inhibitors. This order exposes the non-monotonous response of the brain metabolism to increasing inhibition of OADH by the membrane-penetrating esters. All the esters are compared to AP, which must be transported to a cell through a protein channel or transporter. The transport is limited, compared to the membrane diffusion of the esters. However, inside a cell, AP is ready to act while its esters are to be de-esterified. Thus, in addition to the lipophilicity, there is a balance of several events which may cause quantitative differences in the actions of AP and its esters. For instance, such differences are observed between the effects of AP and TEAP (at 0.02 mmol/kg) and/or TMAP (at 0.1 mmol/kg) on the activities of OADHC or PDHC (Figure 4B). However, most of the metabolic effects of AP administration are similar to those of its esters of high lipophilicity (TEAP) or administered at a high dose (TMAP).

There are two major types of non-monotonous changes across the presented order of the inhibitors (Figure 4). One type manifests a strong perturbation by 0.02 mmol/kg TMAP, followed by a gradual decrease in the perturbation. This type of biphasic change is exemplified by the levels of tryptophan, β-alanine, and malate dehydrogenase (Figures 4B,C). The other type shows more extreme differences between the low TMAP group and all other treatments, as observed for glutamine synthetase and malic enzyme activities, level of β-aminoisobutyrate, and the glutathione redox ratio (Figures 4B,C). Regarding the indicated parameters, the treatment by the low TMAP dose exhibits an effect equally different from any other group. Apart from these obvious types of non-monotonous responses, subtle differences between other groups may be demonstrated by other metabolic parameters. For instance, this is manifested in the activities of OADHC, PDHC, and the level of glutamate.

As a result, increasing the TMAP dose from 0.02 to 0.1 mmol/kg decreases the effects, revealing a non-monotonous response of the brain metabolism to the OADH inhibition. Different metabolic effects of the same dose (0.02 mmol/kg) of TMAP or TEAP agree with a better membrane penetration of TEAP, causing its higher intracellular accumulation, compared to TMAP.

### ECG and Behavioral Changes Upon Increasing OADH Inhibition

**FIGURE 5:**
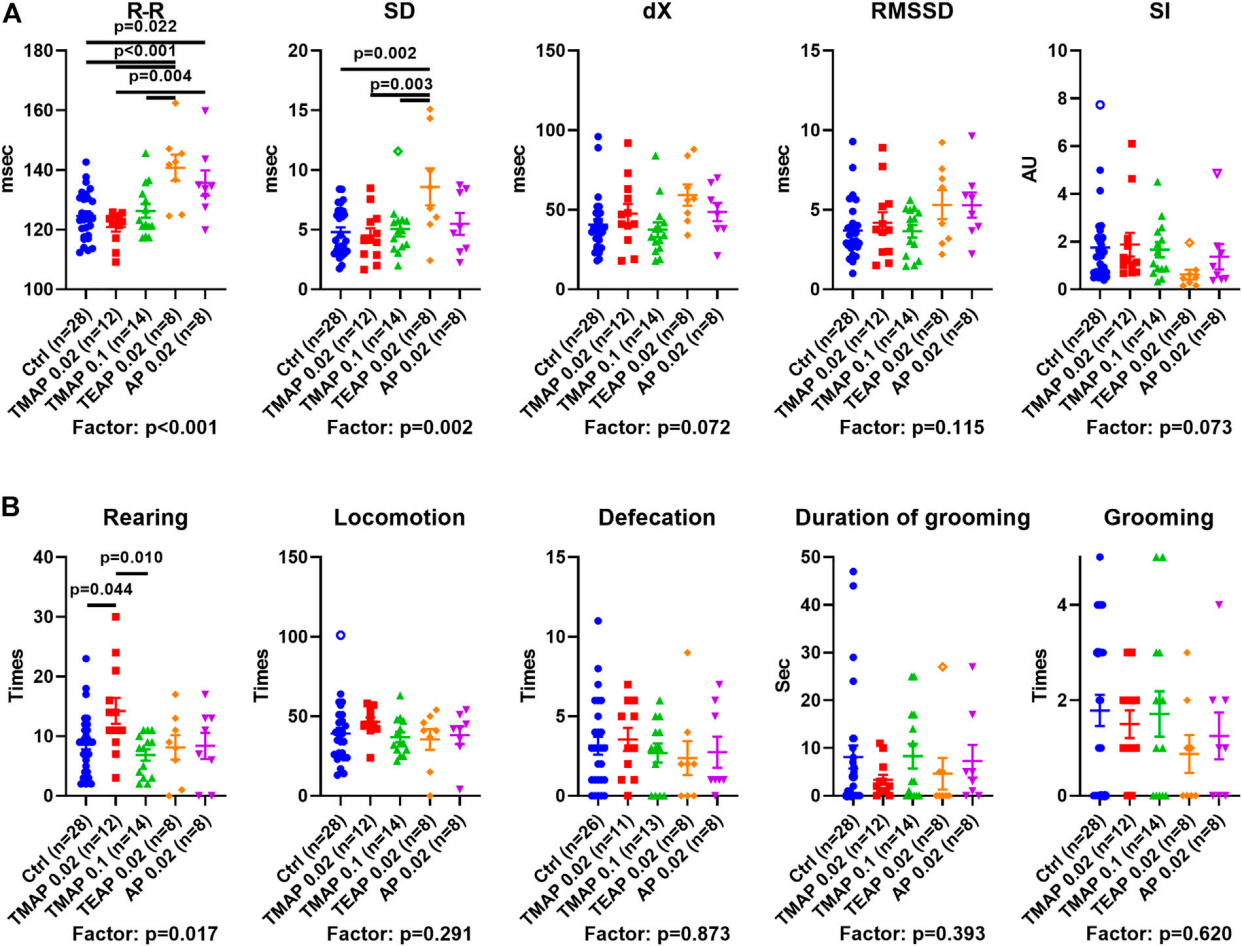
Effects of adipoyl phosphonate (AP), its trimethyl (TMAP), and triethyl (TEAP) esters on physiological parameters. **(A)** ECG parameters of heart regulation (described in Materials and Methods). **(B)** Behavioral parameters are determined in the “open field” test. Significant (*p* ≤ 0.05) differences between the experimental groups, estimated by ANOVA with Tukey’s *post hoc* test, are indicated on the graphs. The ANOVA *p*-values for the treatment factor significance are shown below the graphs. Hollow points correspond to outliers according to the iterative Grubb’s test, excluded from statistical analysis The number of animals in experimental groups (*n*) is indicated including outliers. AU—arbitrary units.

Physiological responses of the rats with perturbed OADH function are presented in Figure 5 in the same order of the treatment groups as in Figure 4. The data show that the R–R interval and its SD are elevated by TEAP and AP (Figure 5A). Thus, a higher level of OADH inhibition affects heart regulation. Behavioral parameters (Figure 5B) reveal an increase in rearing after administration of the low TMAP dose. Together with no changes in the anxiety indicators, such as defecation, grooming (Figure 5B), and stress index of ECG (SI in Figure 5A), the elevated rearing suggests that a low level of OADH inhibition increases exploratory activity.

## Discussion

In this work, we have demonstrated specific regulation of the rat brain metabolism by the OGDH- or OADH-directed inhibitors SP or AP *in vivo*. The data are in good accordance with our earlier study pointing to the selective action of these inhibitors *in vitro* (Artiukhov et al., 2022) and in cultured cells (Artiukhov et al., 2020). At the same dose of the inhibitors and their ethyl esters (0.02 mmol/kg), the brain metabolic profiles exhibit opposite responses to SP/TESP *vs*. AP/TEAP, discriminating specific actions of the OGDH- and OADH-directed phosphonates (Figure 2). These results are in good accordance with existence of structural determinants promoting the binding of SP to OGDH and AP to OADH, as revealed earlier (Artiukhov et al., 2021).

Remarkably, the brain carnosine levels are reduced by the OADH inhibitors only (Car, Figure 2), associated with concomitant accumulation of the carnosine precursor β-alanine (Figure 2A). The findings link the brain metabolism of carnosine, known to have an antioxidant significance in protection of the brain cells from peroxynitrite damage (Nicoletti et al., 2007), to the OADH function.

Specific metabolic action of the OGDHC- and OADHC-directed inhibitors is translated into specific differences in physiological responses to the inhibitors: whereas SP/TESP increases the animal anxiety, the same doses of AP/TEAP increase R–R intervals of ECG (Figure 3). Regarding the ECG effects of AP/TEAP observed in this work, it is worth noting that a recent study on the knockout of the OADH-encoding *DHTKD1*, added by the genome-wide association of the common human variants of *DHTKD1,* found that the gene is linked to cardiovascular risks (Wang et al., 2021). These independent findings are in good accordance with our results on the AP/TMAP effects on ECG (Figures 3, 5).

A comparative study of the trimethylated and triethylated esters of AP reveals significantly different actions. The efficiency of intracellular formation of the inhibitory species (AP) by esterases, as well as inhibitory action of the monoesters of the phosphonate group, known for the phosphonate analogs of 2-oxo acids (Bunik et al., 1992; Biryukov et al., 1996; Bunik et al., 2005), may contribute to the difference. We show that the metabolic and physiological effects of different esters and doses of TMAP correlate with the lipophilicity of the compounds, which is higher for the triethyl than trimethyl esters. Thus, the lipophilicity of the ester group of the phosphonate analogs of 2-oxo acids that determines membrane diffusion of the pro-inhibitors obviously has a significant contribution to their metabolic and physiological action. The similarity of the effects of the high dose of TMAP and the low dose of TEAP (Figure 4) supports this assumption. These actions are rather different from that of TMAP at 0.02 mmol/kg. In particular, specific indicators of the OGDHC inhibition, such as the activities of OGDHC and malate dehydrogenase, are increased at the low TMAP dose, similar to their increases after administration of SP/TESP. However, a simultaneous increase in the glutamine synthetase activity and no change in the glutamate levels (Figure 4C) indicate that the metabolic action of the low TMAP dose differs from that of SP/TESP. The difference is supported by the effect of the low TMAP dose on the exploratory activity of animals (increased rearing in Figure 5B), not observed after administration of SP/TESP (Figure 3).

Thus, increased levels of OADH inhibition may be achieved through elevation of either the TMAP dosage or the lipophilicity of the ester (TEAP instead of TMAP). The brain response to this increase of the OADH inhibition is exemplified by the action of TMAP and TEAP on the brain levels of tryptophan (Figure 4), whose degradation intermediate 2-oxoadipate is irreversibly oxidized by OADHC. Administration of TMAP at 0.02 mmol/kg increases the brain tryptophan level, which is in good accordance with the OADHC inhibition perturbing the tryptophan degradation. However, further increasing TMAP dosage to 0.1 mmol/kg abrogates the effect of its lower dose, almost returning the tryptophan level to the control one. This reversal of the effect may correspond to the network adaptation to decreased tryptophan consumption—for example—through the decreased intracellular supply of tryptophan. The further decrease in the brain levels of tryptophan, observed upon the TEAP and AP treatments, may manifest a more profound inhibition of the tryptophan supply to the brain where the tryptophan degradation is dysregulated more, than upon the treatment with the less efficient TMAP.

Among the common systemic effects on the metabolic network that occur upon the administration of either SP/TESP or AP/TEAP the downregulation of PDHC is worth noting (Figure 2A, cluster 3). The finding suggests that perturbations of mitochondrial metabolism of amino acids by inhibiting either OGDHC or OADHC converge on regulation of PDHC. This common consequence of the OGDH and OADH inhibition may be linked to the role of PDHC in production of acetyl-CoA required for turnover of the tricarboxylic cycle and central role of the cycle in mitochondrial metabolism of amino acids. However, different metabolic states of the brain are achieved upon downregulation of OGDHC or OADHC. This is obvious from the different reactivities of the major part of the assessed metabolic indicators (Figure 2A), further supported by different physiological outcomes of the OGDHC and OADHC inhibition (Figure 3). When OGDHC function is strongly perturbed, the metabolism switches to a state characterized by a lower level of the OGDHC activity and decreased glutathione redox status in the brain, compared to the unperturbed metabolism (Artiukhov et al., 2022). When OADHC function is strongly perturbed, the switch involves decreased levels of tryptophan and malate dehydrogenase activity (Figure 4). Along with the resulting difference in the physiological impact (Figure 3), multiple lines of our data support the selective action of the studied phosphonates *in vivo*.

## Conclusion

AP and its esters are selective inhibitors of the *DHTKD1*-encoded 2-oxoadipate dehydrogenase, while SP and its esters selectively inhibit the *OGDH*(*L*)-encoded 2-oxoglutarate dehydrogenase. Methyl esters of the phosphonates are less lipophilic than the ethyl esters which decreases the intracellular accumulation of the methyl *vs*. ethyl phosphonates through membrane diffusion.

## Data Availability

The raw data supporting the conclusion of this article will be made available by the authors, without undue reservation.

## Glossary

aABA: α-aminobutyrate
Ans: anserine
AP: adipoyl phosphonate
bAiBA: β-aminoisobutyrate
bAla: β-alanine
Car: carnosine
Cit: citrulline
CysThi: cystathionine
DHTKD1: dehydrogenase E1 and transketolase domain-containing protein 1
dX: range of R–R interval
EA: ethanolamine
gABA: γ-aminobutyrate
GDH: glutamate dehydrogenase
GS: glutamine synthetase
GSH: reduced glutathione
GSSG: oxidized glutathione
MDH: malate dehydrogenase
ME: NADP^+^-dependent malic enzyme
OADH: 2-oxoadipate dehydrogenase
OADHC: 2-oxoadipate dehydrogenase complex
OGDH: 2-oxoglutarate dehydrogenase
OGDHC: 2-oxoglutarate dehydrogenase complex
Orn: ornithine
PDHC: pyruvate dehydrogenase complex
R–R: average R–R interval;
RMSSD: root mean square of successive differences in R–R
SD: standard deviation of R—R interval
SI: stress index
SP: succinyl phosphonate
TEAP: triethyl ester of adipoyl phosphonate
TESP: triethyl ester of succinyl phosphonate
TMAP: trimethyl ester of adipoyl phosphonate
