# Supplementary material for: Administration of Phosphonate Inhibitors of Dehydrogenases of 2-Oxoglutarate and 2-Oxoadipate to Rats Elicits Target-Specific Metabolic and Physiological Responses
Source: Front Chem. 2022 Jun 20;10:892284. doi: 10.3389/fchem.2022.892284 (PMC9252169; doi:10.3389/fchem.2022.892284)
Supplement: Supplementary file 1 [file DataSheet1.docx]

**A**

**B**

**C**

**Supplementary Figure 1. NMR spectra of TEAP.** The ^1^H (A), ^31^P (B) and ^13^C (G) spectra of TEAP were obtained after the synthesis.

**Supplementary Figure 2. IR spectra of TEAP (neat).**
